# Formation of Fluorine Vacancy (FV) Centers in Diamond

**DOI:** 10.3390/ma19030494

**Published:** 2026-01-26

**Authors:** Anand B. Puthirath, Jacob Elkins, Harikishan Kannan, Alyssa Horne, Jia-Shiang Chen, Hao Zhang, Valery N. Khabashesku, Abhijit Biswas, Xiang Zhang, A. Glen Birdwell, Tony G. Ivanov, Ulrich Kentsch, Shavkat Akhmadaliev, Robert Vajtai, Xuedan Ma, Aditya D. Mohite, Ranjit Pati, Pulickel M. Ajayan

**Affiliations:** 1 Department of Materials Science and Nanoengineering, Rice University, Houston, TX 77005, USA; jle2@rice.edu (J.E.); harikishan.kannan@gmail.com (H.K.); abhijit.biswas@rice.edu (A.B.); xiang.zhang@rice.edu (X.Z.); robert.vajtai@rice.edu (R.V.); xuedan.ma@rice.edu (X.M.); 2 Department of Physics, Michigan Technological University, Houghton, MI 49931, USA; alyssaho@mtu.edu; 3 Center for Nanoscale Materials, Argonne National Laboratory, Lemont, IL 60439, USA; chen.jiashiang@gmail.com; 4 Center for Molecular Quantum Transduction, Northwestern-Argonne Institute of Science and Engineering, Northwestern University, Evanston, IL 60208, USA; 5 Department of Chemical and Biomolecular Engineering, Rice University, Houston, TX 77005, USA; hz64@rice.edu (H.Z.); aditya.d.mohite@rice.edu (A.D.M.); 6 Applied Physics Program, Smalley-Curl Institute, Rice University, Houston, TX 77005, USA; 7 DEVCOM Army Research Laboratory, Electromagnetic Spectrum Sciences Division, Adelphi, MD 20783, USA; anthony.g.birdwell.civ@army.mil (A.G.B.); tony.g.ivanov.civ@army.mil (T.G.I.); 8 Ionenstrahlzentrum/Ion Beam Center (IBC), Helmholtz-Zentrum Dresden—Rossendorf e.V. (HZDR), Bautzner Landstr. 400, 01328 Dresden, Germany; u.kentsch@hzdr.de (U.K.); akhmadal@hzdr.de (S.A.)

**Keywords:** diamond, color centers, FV center, photoluminescence, DFT

## Abstract

Diamond has been extensively examined as an appealing material for use in quantum optics and quantum information processing owing to the existence of various classes of optically active defects, referred to as “color centers,” which can be engineered into its crystal structure. Among these defects, the negatively charged nitrogen-vacancy center (NV^−^) stands out as the most prominent type. Despite the progress made, the number of emitters characterized by reproducible fabrication processes within the desired spectral range at room temperature, with limited or no damage to the parent diamond lattice, remains restricted. Herein, we are proposing for the first time the creation of the FV^−^ center in diamond via low-energy implantation, which is particularly interesting as it possesses characteristic light absorption and magnetic properties similar to NV^−^ centers. The low-energy ion-implanted FV centers in diamond show more desirable optical emission properties at room temperature (RT). Additionally, as per DFT calculations, the flat bands near the Fermi energy indicate dominant electron–electron interactions, an important prerequisite for observing emergent behavior as seen in systems such as twisted bi-layer graphene. Consequently, as-developed new luminescent defects such as Fluorine Vacancy Centers (FV) with desirable spectral and quantum emission properties would be a significant breakthrough in diamond-based quantum materials.

## 1. Introduction

Diamond has been extensively studied as an attractive material for applications in quantum optics and quantum information processing [1]. This is primarily due to the presence of various classes of optically active defects, commonly known as “color centers,” that can be intentionally engineered into its crystal structure. Among these defects, the negatively charged nitrogen-vacancy center (NV^−^) stands out as the most prominent type. The NV^−^ center offers key advantages such as photo-stability at room temperature, high quantum efficiency, and unique spin properties, making it highly promising for applications in quantum sensing and computing. The quest for single-photon emitters with specific opto-physical properties, such as a high emission rate and narrow linewidth, has led to the exploration and characterization of alternative optical centers in diamond beyond the NV complex. These alternatives involve group-IV impurities (Si, Ge, Sn, Pb) and noble gases (He, Xe) [2]. When it comes to the synthesis of color centers in diamond, ion implantation emerges as a powerful technique for engineering a diverse range of color centers. It allows for precise control over parameters like ion species, energy, and irradiation fluence, influencing the type, chemistry, and density of defect complexes [3].

Despite the progress made, the number of emitters characterized by a reproducible fabrication process at a desired spectral range at room temperature with limited or no damage to the parent diamond lattice remains limited, and a comprehensive investigation in this field is still underway [4]. Consequently, the development of new luminescent defects, for instance, Fluorine Vacancy (FV) Centers with desirable spectral and quantum emission properties through high-energy implantation of specific ion species, remains a pivotal strategy for advancing the field, although such an approach leads to undesirable defects and graphitization of the parent diamond lattice [5]. In this context, we propose a gas-phase fluorine exposure of single-crystal diamond (SCD) wafers and low-energy ion-implantation at room temperature (RT) and elevated substrate temperatures as alternative yet viable approaches to create FV centers in diamond, an alternate analog to NV centers, with more desirable photoemission properties at RT. This would be followed by a systematic characterization of photoluminescence (PL) under varying optical excitation energies and temperatures.

Herein, we report the synthesis of FV centers in single-crystal diamonds near the surface via a facile low-energy ion implantation approach, followed by the characterization of FV center formation via optical, microscopic, chemical, and elemental characterization approaches. The FV centers in the diamond are further understood via DFT calculations in order to understand the formation energy, electronic properties, and absorption and emission spectral frequencies. Finally, the synthesized samples are subjected to rigorous photoluminescence measurements, followed by second-order photo-correlation experiments at RT and low temperatures to study the single-photon emission characteristics (SPE).

## 2. Materials and Methods

**Materials**: General grade single-crystal diamond wafers, which were acquired from ElementSix, Didcot, UK (SC Plate CVD 2.6 × 2.6 mm, 0.25 mm thick, <100> with substitutional impurity concentrations N < 1 ppm, and B < 0.05 ppm), have been employed in the experiments.

**Low-energy ion implantation**: The implantations were carried out on the DANFYS 1090-50 implantation system at the Ion Beam Centre of the Helmholtz-Zentrum Dresden-Rossendorf. The ions were extracted from a high-current ion source “CHORDIS” (magnetic multi-cusp plasma discharge ion source) and separated by a 90° analyzing magnet. The ion beam was focused by an Einzel lens and a quadrupole triplet and was scanned over the sample at a frequency of 1 kHz by an X-Y scanner to ensure a homogeneous ion distribution. The beamline is equipped with a neutral trap to prevent neutral particles from hitting the sample. The ions with an energy of 1.5 keV were implanted with a flux of 2 × 10^11^ s^−1^cm^−2^ at 200 °C under normal incidence. The fluence was 1 × 10^15^ cm^−2^.

**Cryogenic Photoluminescence Spectroscopy**: The cryogenic micro-photoluminescence (μ-PL) spectroscopy was measured based on a lab-built confocal microscopy system. The sample was photo-excited using a supercontinuum pulsed laser (repetition rate 78 MHz, NKT Photonics, Birkerød, Denmark) selected at 480 nm. The laser was focused onto the sample through a 50× objective (0.42 NA) with ~1 μm beam size, yielding an excitation intensity of 6.4 × 10^4^ W/cm^2^ (0.5 mW laser power). The PL data was collected from 500 to 950 nm (1.3–2.4 eV) by a spectrometer (Andor Kymera) with a 400 lines/mm grating and a CCD camera (Andor iDus 416). During PL measurement, the sample was kept under vacuum (10^−5^ torr) in a closed-cycle cryostat (Advanced Research Systems) and maintained at cryogenic temperature (T = 6.5 K).

**Time-resolved PL spectroscopy and second-order photon-correlation measurements**: The samples were loaded into a home-built confocal laser scanning microscope. A microscope objective (40×, NA = 0.95) was used to focus the laser excitation beam (440 nm) onto the samples. PL from the samples was collected by the same objective and directed to either a spectrograph for measurements or single-photon counting detectors with time-correlated photon-counting electronics (PicoQuant). For second-order photon-correlation measurements, the collected PL was spectrally filtered by a combination of long-pass filters (475 nm and 710 nm) and then split by a 50:50 beam splitter and focused onto two single-photon counting detectors in Hanbury Brown-Twiss (HBT) geometry.

**DFT Calculations**: We used the Vienna Ab Initio Simulation package (VASP) [6,7,8,9] with projector-augmented wave formalism to carry out the spin-polarized Density Functional Theory (DFT) calculations. A generalized gradient approximation (GGA) with the Perdew–Burke–Ernzerhof (PBE) exchange–correlation functional [10] was used. The cutoff energy for the basis of the plane wave was 600 eV. To overcome the size effects, we created a reasonably large supercell with 216 atoms by extending the cubic diamond unit cell along all three directions. Subsequently, a negatively charged nitrogen-vacancy defect was introduced into the supercell by substitutional doping of negatively charged nitrogen with a vacancy at the nearest neighbor site. To find the ground state configurations, we optimized the lattices (both pristine diamond and NV^−^ defect structure) with ionic relaxation using the conjugate gradient algorithm [11]. All ions in the supercell were allowed to relax until the residual force at each atom was less than 0.01 eV/Å. The energy convergence was set to 1.0 × 10^−7^ eV during the self-consistent electronic structure calculation. The Brillouin zone was sampled using a 5 × 5 × 5 Monkhorst–Pack k-point mesh. A similar procedure was followed to create a negatively charged Fluorine Vacancy Center in the diamond. To help identify the inter-band transition, we calculated the absorption coefficients using the effective single-particle DFT method.

The defect formation energy for the NV^−^ (or FV^−^) center was calculated using the following formula [12,13,14,15,16,17]:

(1) $$E_{Form}\;=E_{D}-\;E_{C}^{Bulk}+\;\frac{2}{216}E_{C}^{Bulk}\;-E_{A}\;-\left(E_{C}^{Bulk}\;-\;E_{C}^{+}\right)\;-\;△E_{f}+E_{Corr}$$

where $E_{Form}$ is the defect formation energy; $E_{D}$ is the total energy associated with the host diamond structure with the charge defect; $E_{C}^{Bulk}$ is the total energy of the host diamond without defects; $E_{A}$ is the energy associated with a single N (or F) atom in the gas phase for the NV^−^ (or FV^−^) center; $E_{C}^{+}$ is the energy of the pristine diamond with a hole; and $△E_{f}$ is the energy width between the maximum of valence band and the Fermi energy of the host pristine diamond. $E_{Corr}$ is the correction term that takes into account energy-band-edge corrections due to the approximate nature of the exchange-correlation functional used in the DFT, as well as unintended electrostatic interactions between the defect and its images in the periodic supercell.

## 3. Results and Discussion

### 3.1. Synthesis of FV Centers in Single-Crystal Diamond via Low-Energy Ion Implantation

Multiple methods of doping diamonds have been developed over the years since the realization of their high potential in electronic devices, with plasma CVD and ion implantation being some of the most prominent methods. But in the case of diamond, high-energy ions often lead to lattice damage and amorphization in the crystal, consequently causing adverse effects for the optical and semiconductor applications. While some of this damage can be rectified by thermal annealing after doping, it often leads to the formation of graphite, and careful optimization is necessary.

**Figure 1:**
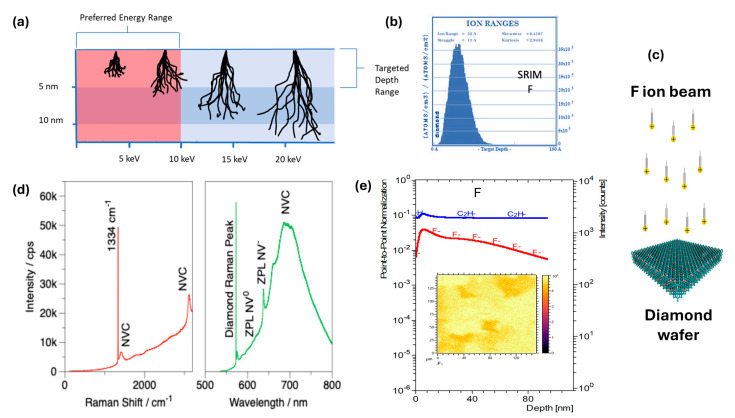
Low-energy ion implantation and characterization: (**a**) Schematic showing the proposed low-energy implantation process, where the ions can be placed on the surface region without damaging the parent lattice. (**b**) The Stopping and Range of Ions in Matter (SRIM) projection of the distribution of implanted F ions in the diamond lattice with respect to depth. (**c**) Graphic presentation of the process of ion implantation into the surface of a diamond wafer. (**d**) The Raman and PL spectra of the as-implanted sample. (**e**) TOF-SIMS depth profiling for C and F elements and mapping of F across the diamond wafer.

Low-energy ion implantation is expected to introduce dopant ions to desired depth levels without damaging the diamond structure and enable FV centers in diamond with competing optical and electronic properties compared to the widely explored wet chemical approaches and high-energy ion implantation (>30 keV). The schematic showing the variation in the implantation depth against implantation energy is shown in Figure 1a. Based on the preliminary tests and SRIM calculations (Figure 1b), the proposed parameters for F ion implantation are 1.5 keV energy with 1 × 10^15^ Fluence (cm^−2^) and Flux 1—2.2 × 10^11^ Flux (cm^−2^/s) at substrate temperatures of 200 °C. The schematic showing the implantation procedure is also depicted in Figure 1c. Raman, photoluminescence, and secondary ion mass spectroscopy (SIMS) studies performed on as-synthesized FV centers in diamond are provided in Figure 1d,e, respectively. The pumping wavelength used was 480 nm using a monochromatic pulsed laser with ~1 μm beam size, at 0.5 mW laser power. It can be inferred that the graphitization is negligible for the low-energy implanted sample, and the implantation depth is as predicted by the SRIM calculation. It is worth noting that, although implanted species are found in the diamond sample, no characteristic emission is observed on the as-implanted sample.

### 3.2. Activation of Implanted Species in Diamond Wafer and Photoluminescence Studies

**Figure 2:**
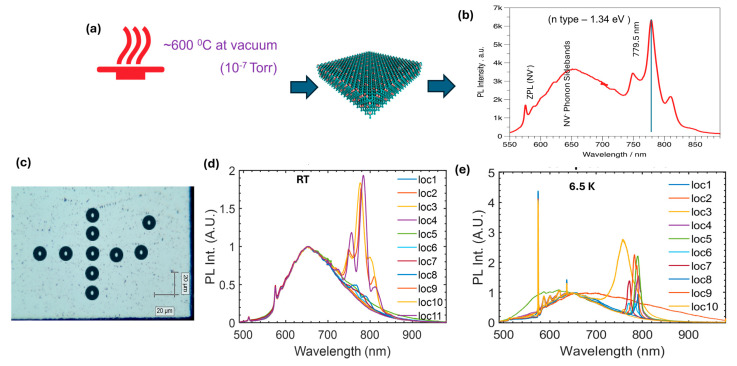
The activation process of the F-implanted diamond wafer and photoluminescence analysis. (**a**) Schematic showing the annealing conditions of the implanted wafer, and the F-vacancy-activated diamond crystal shown in dark blue color. (**b**) The single-point photoluminescence spectra of the activated FV center. (**c**) Systematic scanning to detect the formation of an FV center in the diamond wafer. (**d**,**e**) Corresponding PL spectra at room and (**e**) cryogenic temperatures.

The as-F implanted diamond wafer is subjected to 600 °C annealing for four hours under high vacuum conditions (10^−7^ Torr, Figure 2a), and the obtained wafer is subjected to detailed photoluminescence studies at room as well as cryogenic (~6.5 K) temperatures (Figure S3). The range of the PL data was collected from 500 to 950 nm (1.3–2.4 eV) at room temperature under a vacuum of 10^−5^ Torr. There is a broad emission observed within the 530–950 nm spectral region (1.3–2.3 eV) centered around ~700 nm (Figure 1d) from the pristine SCD sample, which is attributed to the nominal concentrations of substitutional nitrogen present in the SCD substrate within the range of 5–100 ppb levels. Interestingly, while investigating the emission spectra from the low-energy implanted wafer at room temperature, new additional features were observed across a high wavelength region that could possibly be attributed to fluorine defect or vacancy centers (F-V). The single narrow emission lines centered at 780 nm with shoulder peaks can be seen in Figure 2b. This clearly differs from conventional defect centers such as Silicon-Vacancy (Si-V) and NV centers [18]. The FV center regions (Figure 2c) were scanned to check the distribution of FV center formation in the implanted SCD wafer and the PL emission spectra, taken from several different locations at room temperature and cryogenic temperature, respectively, and are displayed in Figure 2d,e. At the cryogenic temperature, the PL emission is found to be further narrowed, and the shoulder peaks are suppressed owing to the suppression of phonon modes that influence the emission spectrum. Although the FV center formation is not uniform across the surface, we observed several emission centers (>100 μm area) across the synthesized sample surfaces.

### 3.3. Coherent Light Emission Studies of FV Center in Diamond

**Figure 3:**
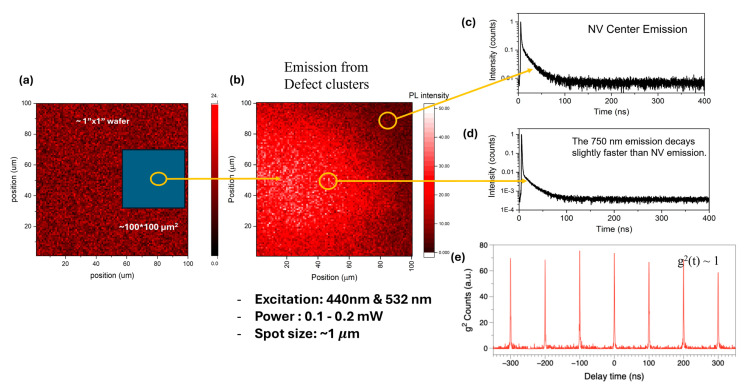
Time-resolved photoemission studies at room temperature: (**a**) the scanning matrix with area 100 × 100 μm^2^; (**b**) the photograph showing the emission from FV centers; (**c**,**d**) the decay characteristics of the emission from NV centers and FV centers. (**e**) The results of second-order autocorrelation emission studies showing the coherent emission characteristics.

Observing the narrow emission at a higher wavelength range compared to the most studied NV centers, we employed the Hanbury Brown–Twiss (HBT) interferometer to study the emission characteristics of FV centers formed in diamond compared with the pristine wafer that contains a marginal amount of NV centers for possible single-photon emission characteristics. The experiment conditions and the PL scanning image of a 100 × 100 μm^2^ area region are displayed in Figure 3a,b, respectively. The region with the activated FV centers appears to be brighter than the rest. Comparing time-resolved PL decay curves from the FV centers (Figure 3d) with those of NV^−^ centers (Figure 3c), we found that the FV centers decay slightly faster than the NV^−^ centers. Figure 3e shows a representative second-order photo-correlation function measured in the area highlighted in Figure 3d. A long-pass filter was used to select emissions from FV centers and block any residual emissions from NV centers. We observed similar intensities for the center peak at delay time zero and side peaks; namely, no apparent photon-antibunching was detected. We speculate that this is due to the high densities of FV centers excited by the laser beam, which prevents the detection of single defects.

### 3.4. Theoretical Understanding of FV Formation in Diamond and Its Properties

**Figure 4:**
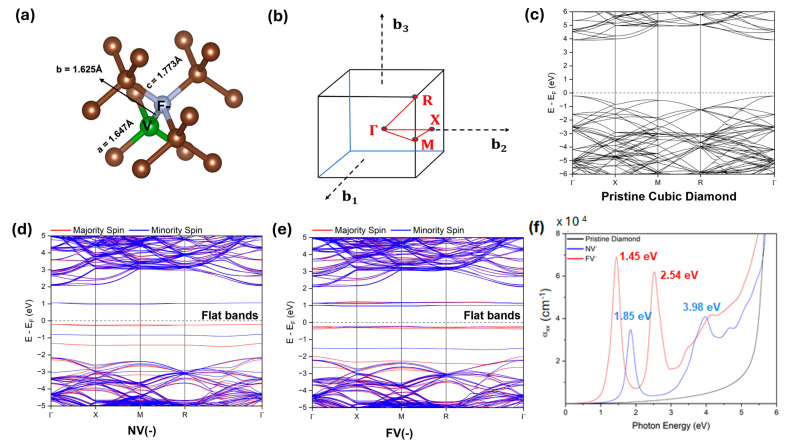
Band structures of cubic diamond, cubic diamond with NV^−^ center, and cubic diamond with FV^−^ center. (**a**) The local atomic-level structural distortion in cubic diamond with FV^−^ center. (**b**) Brillouin zone depicting the high symmetry path in the reciprocal space for electronic band structure calculations. The energy band diagram for (**c**) pristine cubic diamond, (**d**) NV^−^ center, and (**e**) FV^−^ center. The Fermi energy is shown by the dotted line. (**f**) The simulated optical absorption coefficients of pristine cubic diamond, NV^−^, and FV^−^ centers in diamond.

Since the diamond lattice is dense, diffusion of ions is expected to be minimal, and the surface is very unreactive, the formation of active FV centers, with a low temperature (600 °C) activation process via post-annealing, is very surprising. This raises questions about the formation energy of such entities in the diamond lattice, which is not very well understood or explained in the literature. Hence, to understand the system better, we performed detailed DFT simulations and obtained an atomic structure, lattice parameters, formation energy, energy band structures, and absorption coefficients (Figure 4).

The band structure for the pristine diamond and the NV^−^-center was found to be in good agreement with previously reported values [19,20], and the optimized lattice parameters in the supercell structures were found to be 10.71 Å and 10.74 Å, respectively. For the FV^−^ center in Figure 4a, the lattice parameter expands to 10.75 Å. This lattice expansion is also apparent in the bond lengths around defects. Bond lengths between the F and adjacent C atoms are found to be 1.77 Å. This value was significantly higher than the N and adjacent carbon bond distance in the NV^−^ center. However, the distance between F and vacancy is 1.63 Å in FV^−^, which is smaller than the distance between the nitrogen and vacancy (1.69 Å) in NV^−^. Bader charge analysis indicates the localization of negative charge at the F site, as observed at the N-site in the NV^−^ center. However, only ~50% of the negative charge is localized at the F-site. Ignoring the energy correction term in Equation (1) (see Section 2), the defect formation energy was found to be 2.22 eV and 9.37 eV for NV^−^ and FV^−^ centers, respectively. Though the formation energy is relatively higher for the FV^−^ center, as expected, it confirms the plausible formation of FV^−^ defects in the pristine diamond.

Next, we analyzed the energy band structure along the high symmetry path shown in Figure 4b and Figure S1. The cubic diamond was an insulator with a large indirect band gap of 4.12 eV (Figure 4c). The band gap, however, for the cubic diamond was found to be smaller than the experimental gap [21]. This is expected as the GGA approach adopted here has been found to underestimate the gap due to the absence of self-interaction correction; a hybrid approach (HSE06) that includes self-interaction correction partially via using the Hartree–Fock exchange has been reported to correct this discrepancy with the experiment [22,23]. However, this hybrid approach is computationally expensive for the large supercell used here. The NV^−^ (Figure 4d) and FV^−^ (Figure 4e) were found to be ferromagnetic with strong splitting between the spin-up and spin-down states. Furthermore, the energy band gaps were found to be significantly lower in the case of the defect structure compared to pristine diamond. A closer examination indicated near-flat bands in the vicinity of the Fermi energy in the FV^−^ center as observed for the NV^−^ center (Figure S2). This confirms the highly localized nature of the charge defects and justifies the negligible unintended electrostatic interaction between the defect and its periodic images in our supercell approach. It should be noted that flat bands were also reported in the NV^−^ center [24], and our results agree. Flat bands indicate dominant electron–electron interactions in these defect structures, an important prerequisite for observing interaction-driven emergent behavior. It can be probed directly by ARPES or Scanning Tunneling Spectroscopy through observation of van Hove’s singularity.

Since optical spectroscopy acts as a probe for the electronic structure of the material by identifying the inter-band transition, we calculated absorption coefficients using DFT within the effective single-particle picture. In this simplified (static) approach that does not include dynamical screening, the imaginary part of the dielectric function is calculated using an independent-particle approximation, and the Kramers–Kronig relation is used to obtain the absorption coefficients [25]. These results are summarized in Figure 4f. We should note that the excited state cannot be adequately described by a single-particle DFT. A more accurate GW [26,27,28] or hybrid DFT would provide a better understanding of the excited state. However, they are prohibitively expensive for the large supercell calculation used here to mimic the localized defect. Nevertheless, a reasonable understanding of inter-band transition can be obtained from DFT. There were no peaks observed in cubic diamonds for the energy range below 5 eV, confirming the insulating nature of the cubic diamond as observed from the energy band diagram (Figure 4c).

The sharp peak predicted at 1.45 eV in the FV^−^ case indicates the inter-band transition between the spin-majority valence band and conduction band (Figure 4e). The availability of more electronic states closer to the valence band explains more than one state contributing to the strong absorption peak at 1.45 eV. The peak at 2.54 eV could be due to the transition from the low-lying minority spin states (~1.5 eV below the Fermi energy). While comparing the absorption peaks between NV^−^ and FV^−^, we found that FV^−^ is redshifted as observed in the experiment.

## 4. Conclusions

Synthesis of FV centers in a single-crystalline diamond near the surfaces, akin to NV centers via a facile low-energy implantation approach, is reported and found to be reliable and reproducible. This can help create a homogeneous FV emission center across the proximity of diamond wafer surfaces. Various spectroscopic studies revealed the presence of an FV center in diamond, and a time-resolved emission experiment shows its coherent emission characteristic near the communication band. In addition, DFT studies unravel the atomic structure, lattice parameters, absorption coefficients, and band structures, and are found to be akin to the NV centers in diamond. As a future prospect, one needs to explore different chemical environments of FV centers, similar to NV centers in diamond, such as FV^0^, FV^+^, and their magnetic ordering and spin state, for instance, antiferromagnetic nature and spectral emission wavelengths. Such exploration leads to the identification of the tunability of emission wavelength, magnetic sensing behavior, and the possibility of quantum-entangled FV center states, leading to quantum computation and sensing-level applications.

## Data Availability

The original contributions presented in this study are included in the article/Supplementary Materials. Further inquiries can be directed to the corresponding authors.

## Supplementary Materials

The following supporting information can be downloaded at https://www.mdpi.com/article/10.3390/ma19030494/s1, Figure S1: The Atom Decomposed Density of States (DOS) for pristine cubic diamond, NV^−^ and FV^−^ centers are shown in the (a) top, (b) middle, and (c) bottom panels, respectively. The DOS for the majority of spin carriers is presented in the upper section of the plots; the DOS for the minority of spin carriers is presented in the lower section of the plots. The Fermi energy is shown by the dotted line. Figure S2: The magnetization density for the (a) NV^−^ and (b) FV^−^ centers. The dangling bonds of the carbon atoms nearest to the vacancy contribute to the magnetization in the defect structures. The magnetization for NV^−^ and FV^−^ was found to be 1.24 μ_B_ per unit cell. (c,d) depict the charge density due to defects in the NV^−^ and FV^−^ centers, respectively. They were obtained by subtracting the charge density of the cubic diamond with a vacancy defect from the charge density of the NV^−^ and FV^−^ centers, respectively. The red color represents the negative charge density, and the blue color represents the positive charge density. Figure S3: Normalized PL spectra of pristine diamond sample at (a) room temperature and (b) cryogenic temperature (T = 6.5 K), respectively. PL spectra from multiple locations are collected and indicated in different colors. ZPL of NV^−^ and phonon sidebands are detected. No additional spectral feature is observed.
