# Supplementary material for: Formation of Fluorine Vacancy (FV) Centers in Diamond
Source: Materials (Basel). 2026 Jan 26;19(3):494. doi: 10.3390/ma19030494 (PMC12898828; doi:10.3390/ma19030494)
Supplement: Supplementary file 1 [file materials-19-00494-s001.zip › materials-4053640-supplementary.pdf]

Formation of Fluorine Vacancy (FV) Centers in Diamond

Anand B. Puthirath*[1], Jacob Elkins[1], Harikishan Kannan[1], Alyssa Horne[2], Jia-Shiang Chen[3,4], Hao Zhang[5,6], Valery N. Khabashesku*[1], Abhijit Biswas[1], Xiang Zhang[1], A. Glen Birdwell[7], Tony G. Ivanov[7], Ulrich Kentsch[8], Shavkat Akhmadaliev[8], Robert Vajtai[1], Xuedan Ma[1,3], Aditya D Mohite[5], Ranjit Pati*[2], Pulickel M. Ajayan*[1]

[1]Department of Materials Science and Nanoengineering, Rice University, Houston, Texas 77005, USA

[2]Department of Physics, Michigan Technological University, Houghton, MI 49931

[3]Center for Nanoscale Materials, Argonne National Laboratory, Lemont, Illinois 60439, United States

[4] Center for Molecular Quantum Transduction, Northwestern-Argonne Institute of Science and Engineering, Northwestern University, Evanston, Illinois 60208, United States

[5]Department of Chemical and Biomolecular Engineering, Rice University, Houston, Texas 77005, USA

[6]Applied Physics Program, Smalley-Curl Institute, Rice University, Houston, TX, USA.

[7]DEVCOM Army Research Laboratory, Electromagnetic Spectrum Sciences Division, Adelphi, MD 20783, USA

[8] Ionenstrahlzentrum / Ion Beam Center (IBC), Helmholtz-Zentrum Dresden - Rossendorf e.V. (HZDR) Bautzner Landstr. 400 , 01328 Dresden, Germany

Email : anandputhirath@rice.edu, khval@rice.edu, patir@mtu.edu, ajayan@rice.edu

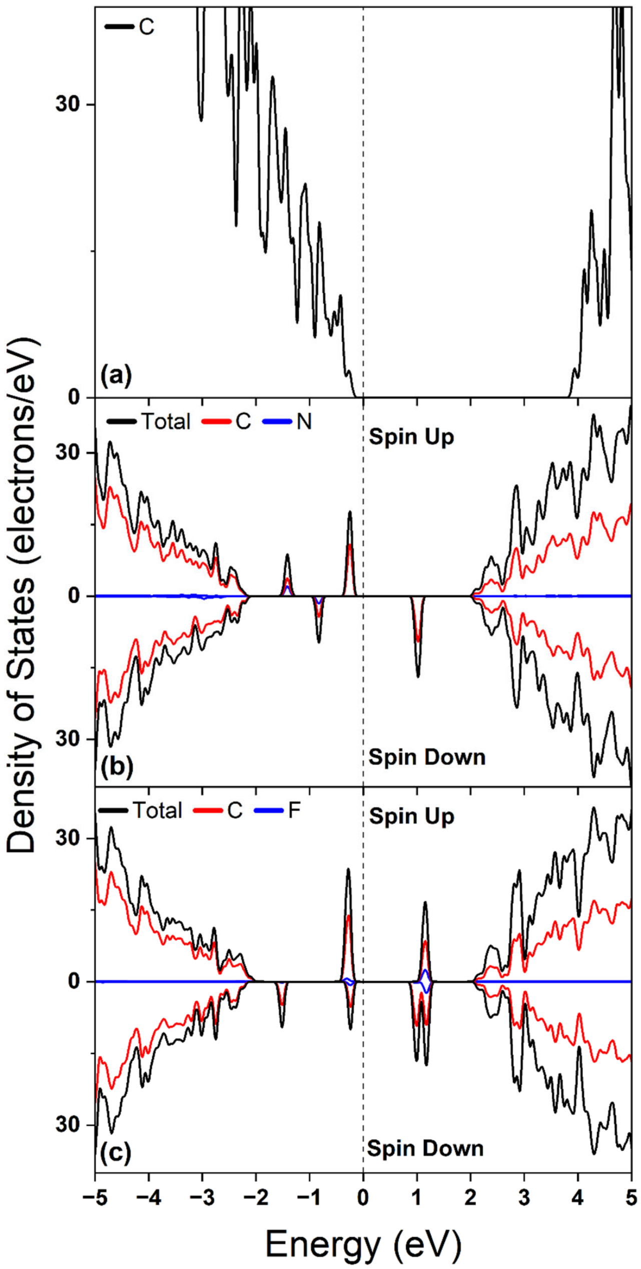

Figure S1. The Atom Decomposed Density of States (DOS) for pristine cubic diamond, NV^- and FV^- centers are shown in the **(a)** top, **(b)** middle, and **(c)** bottom panels, respectively. The DOS for the majority of spin carriers is presented in the upper section of the plots; the DOS for the

minority of spin carriers is presented in the lower section of the plots. The Fermi energy is shown by the dotted line.

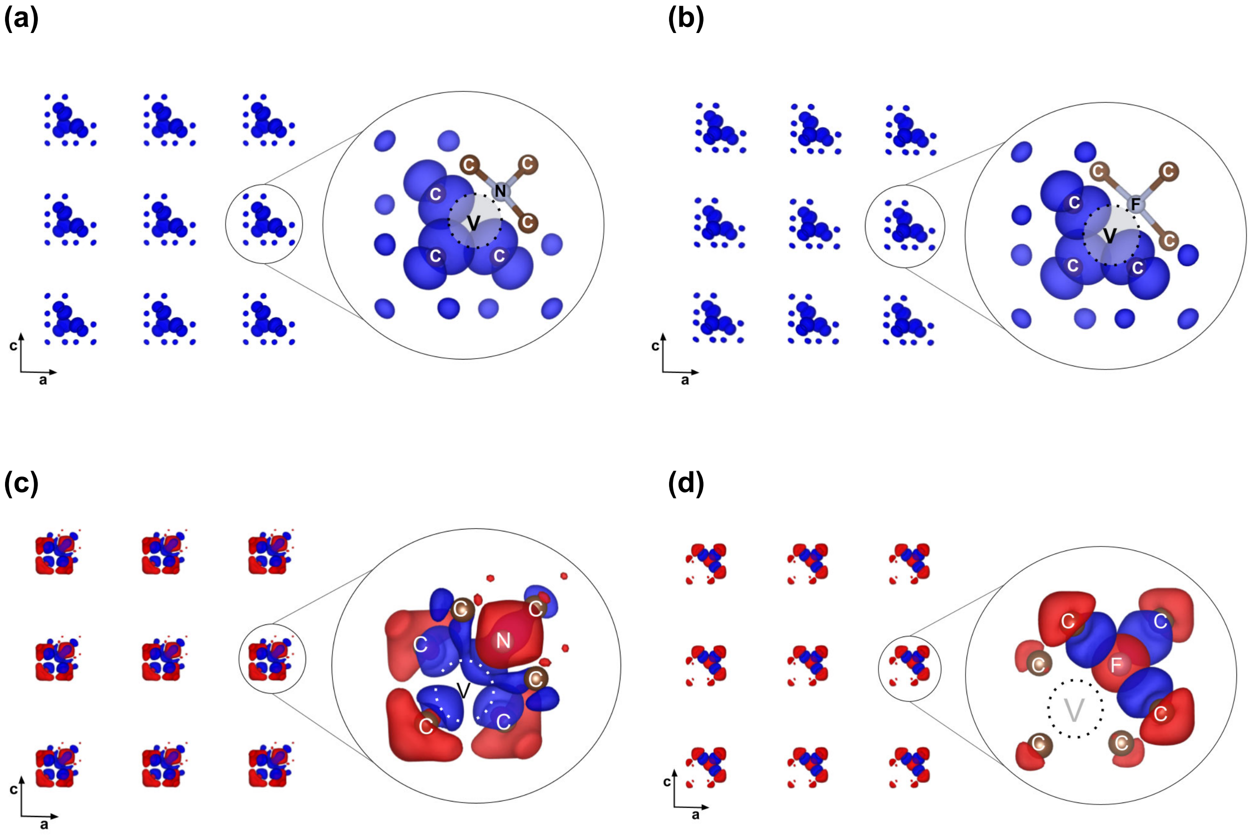

Figure S2. The magnetization density for the **(a)** NV^{-} and **(b)** FV^{-} centers. The dangling bonds of the carbon atoms nearest to the vacancy contribute to the magnetization in the defect structures. The magnetization for NV^{-} and FV^{-} was found to be 1.24 μ_B per unit cell. **(c)** and **(d)** depict the charge density due to defects in the NV^{-} and FV^{-} centers, respectively. They were obtained by subtracting the charge density of the cubic diamond with a vacancy defect from the charge density of the NV^{-} and FV^{-} centers, respectively. The red color represents the negative charge density, and the blue color represents the positive charge density.

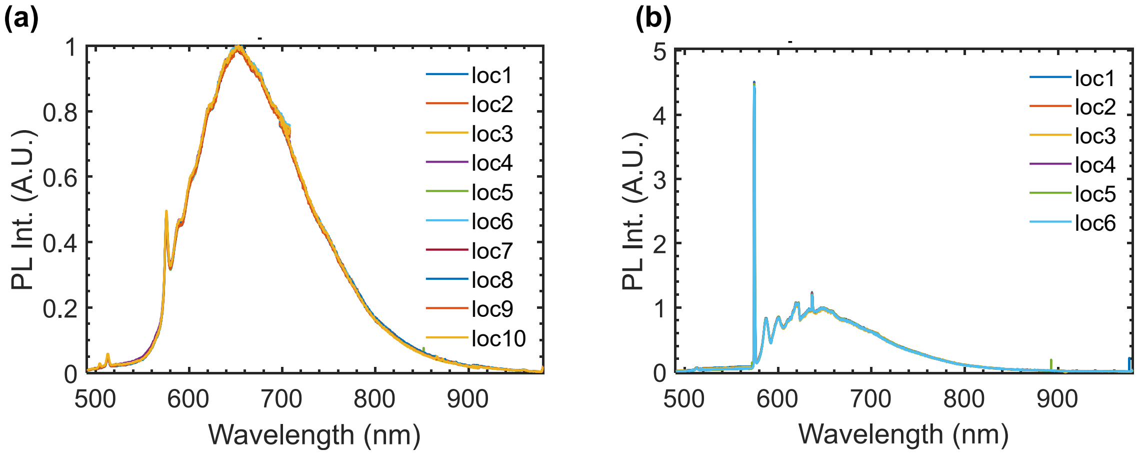

Figure S3. Normalized PL spectra of pristine diamond sample at **(a)** room temperature and **(b)** cryogenic temperature (T = 6.5K), respectively. PL spectra from multiple locations are collected and indicated in different colors. ZPL of NV^- and phonon sidebands are detected. No additional spectral feature is observed.
